# Investigation of olfactory function in a Panx1 knock out mouse model

**DOI:** 10.3389/fncel.2014.00266

**Published:** 2014-09-12

**Authors:** Stefan Kurtenbach, Paige Whyte-Fagundes, Lian Gelis, Sarah Kurtenbach, Émerson Brazil, Christiane Zoidl, Hanns Hatt, Valery I. Shestopalov, Georg Zoidl

**Affiliations:** ^1^Department of Psychology, Faculty of Health, York UniversityToronto, ON, Canada; ^2^Department of Cell Physiology, Ruhr University BochumBochum, Germany; ^3^Department of Ophthalmology, Bascom Palmer Eye Institute, Miller School of Medicine, University of MiamiMiami, FL, USA; ^4^Vavilov Institute of General Genetics, Russian Academy of SciencesMoscow, Russia

**Keywords:** pannexin, Panx1, olfaction, knock out mouse, extracellular ATP, electroolfactogram, behavior

## Abstract

Pannexin 1 (Panx1), the most extensively investigated member of a channel-forming protein family, is able to form pores conducting molecules up to 1.5 kDa, like ATP, upon activation. In the olfactory epithelium (OE), ATP modulates olfactory responsiveness and plays a role in proliferation and differentiation of olfactory sensory neurons (OSNs). This process continuously takes place in the OE, as neurons are replaced throughout the whole lifespan. The recent discovery of Panx1 expression in the OE raises the question whether Panx1 mediates ATP release responsible for modulating chemosensory function. In this study, we analyzed pannexin expression in the OE and a possible role of Panx1 in olfactory function using a Panx1^−/−^ mouse line with a global ablation of Panx1. This mouse model has been previously used to investigate Panx1 functions in the retina and adult hippocampus. Here, qPCR, *in-situ* hybridization, and immunohistochemistry (IHC) demonstrated that Panx1 is expressed in axon bundles deriving from sensory neurons of the OE. The localization, distribution, and expression of major olfactory signal transduction proteins were not significantly altered in Panx1^−/−^ mice. Further, functional analysis of Panx1^−/−^ animals does not reveal any major impairment in odor perception, indicated by electroolfactogram (EOG) measurements and behavioral testing. However, ATP release evoked by potassium gluconate application was reduced in Panx1^−/−^ mice. This result is consistent with previous reports on ATP release in isolated erythrocytes and spinal or lumbar cord preparations from Panx1^−/−^ mice, suggesting that Panx1 is one of several alternative pathways to release ATP in the olfactory system.

## Introduction

Genomes of higher vertebrates contain three pannexin genes (Panx1, Panx2, and Panx3) that share homologies with invertebrate gap junction proteins named innexins (Baranova et al., 2004) and membrane topologies similar to vertebrate connexins, a novel class of integral membrane glycoproteins. Unlike connexins, pannexins function as unopposed channels (Sosinsky et al., 2011) that open through various stimuli like activation of purinergic receptors and high intracellular calcium (Locovei et al., 2006), cellular stretch (Bao et al., 2004), high extracellular potassium levels (Silverman et al., 2009), and depolarization (Bruzzone et al., 2003; Pelegrin and Surprenant, 2006). Pannexins are known to be expressed in sensory systems like the cochlea (Wang et al., 2009), retina (Dvoriantchikova et al., 2006; Zoidl et al., 2008), taste buds (Huang et al., 2007), and olfactory epithelium (OE) (Zhang et al., 2012).

Panx1 has moved into the focus of purinome research due to the ability to form large channel pores capable of conducting big molecules like ATP. The presence of pannexins in different chemosensory systems raises the general question of whether they may contribute to sensory perception by releasing ATP and modulating purinergic signaling. Perception of sensory stimuli also involves purinergic signaling at various levels: ATP can influence cochlear function through multiple mechanisms, including modulation of hearing sensitivity, sound transduction, neurotransmission, and even influencing gap-junctional coupling (Bobbin and Thompson, 1978; Muñoz et al., 1995; Zhu and Zhao, 2012). ATP release plays a primary role in signal transmission of taste cells to afferent nerve fibers (Finger et al., 2005) and a similar mechanism seems to hold true for keratinocytes (Azorin et al., 2011). In the retina, purinergic signals act as neuro- and gliotransmitters, most likely modulating retinal responses on various levels (Prochnow et al., 2009; Wurm et al., 2011; Vessey and Fletcher, 2012). In the OE, extracellular ATP elicits increases in [Ca^2+^]_i_ in both olfactory sensory neurons (OSNs) and sustentacular cells (SCs), leading to a suppression in odor responsiveness (Hegg et al., 2003). ATP also evokes inward currents and increased [Ca^2+^]_i_ in sensory neurons in the vomeronasal organ (VNO), by activation of P2X receptors (Vick and Delay, 2012).

The introduction of several Panx1 knock out animal models, with distinct genetic ablation strategies (Bargiotas et al., 2011, 2012; Dvoriantchikova et al., 2012; Hanstein et al., 2013), has initiated ample opportunities to investigate Panx1 functions from genes through to systems and behavioral outcomes. Since sensory inputs to the CNS can be altered in Panx1^−/−^ mice, as shown recently for the retina (Kranz et al., 2013; Vroman et al., 2014), it was hypothesized that similar changes could be found in other sensory organs. In this study, we characterize the impact of genetic ablation of Panx1 in the olfactory system. Expression and localization studies demonstrated Panx1 expression in the OE. Surprisingly, Panx1 was mainly localized in OE axon bundles and not at the apical surface of the cilia, where the olfactory receptors are present and the olfactory signal transduction cascade is initiated. The OE of Panx1^+/+^ and Panx1^−/−^ mice showed no altered response to odors in electroolfactogram (EOG) measurements, suggesting that Panx1 is dispensable for acute olfactory function. However, a quantitative comparison of extracellular ATP concentration revealed significant differences between the two genotypes after exposure to potassium gluconate. Further, behavioral outcomes revealed small, but significant abnormalities in the processing of olfactory information, accompanied by a higher mobility of Panx1^−/−^ mice. Our data do not support a prominent role of Panx1 channels in olfaction, and it was concluded that the behavioral abnormalities observed in Panx1^−/−^ mice derive from alterations of integrating neuronal processes, as observed in the hippocampus. However, our data suggest that Panx1 is one of several alternative pathways to release ATP in the olfactory system and that dissecting these pathways will be a critical step to define the exact role(s) of Panx1 in sensory systems.

## Methods

### Panx1 knockout mice

Panx1^+/+^ mice (Panx1fl/fl) with three LoxP consensus sequences integrated into the Panx1 gene and knockout mice with global loss of Panx1 (Panx1^−/−^, CMV-Cre/Panx1) were described previously (Gründken et al., 2011; Dvoriantchikova et al., 2012; Prochnow et al., 2012). Handling and housing of animals used in this study was performed in compliance with the German Animal Rights law and approved by the Landesamt für Natur, Umwelt und Verbraucherschutz Nordrhein-Westfalen, Germany (Permission No. 50.8735.1 Nr. 100/4) and formal approval by the Animal Care Committee (York University, Canada). Adult male mice (4–8 months of age) were housed individually 1 week prior to, and during, behavior testing.

### *In-situ* hybridization (ISH)

Digoxigenin (dig)-labeled sense and antisense cRNA probes were prepared from a full length Panx1 cloned into the pcDNA3 plasmid as described previously (Ray et al., 2006). After linearization of the plasmid, sense and antisense cRNA probes were transcribed using T7 and SP6 RNA polymerase with dig-RNA labeling mix (Roche, Germany). The ISH was performed as described (Larsson et al., 2004) with minor modifications. OE from P7 mice were dissected and immediately embedded in tissue freezing medium (Leica, Germany) at −30°C and cryostat sections (12 μm) were cut immediately. Slides were subsequently fixed in 4% paraformaldehyde in PBS at 4°C for 20 min, washed in PBS and acetylated by a 15 min treatment in 0.1 M triethanolaminhydrochloride solution with 0.25% acetic anhydride on a stir plate. Sections were rinsed in 2× SSC (30 mM NaCl and 3 mM sodium citrate) and prehybridized in hybridization buffer (50% formamide, 5× SSC, 5× Denhardts' solution, 2.5 mM EDTA, 50 μg/ml heparin, 250 μg/ml tRNA, 500 μg/ml salmon sperm DNA, and 0.1% Tween-20) for 1 h at 55°C. Riboprobes were added to the hybridization buffer (50 ng in 200 μl hybridization buffer), denaturized at 80°C for 2 min and applied to sections. Sections were incubated over night at 55°C for hybridization. Post-hybridization, slides were washed with 0.2× SSC for 1 h and then with 0.1× SSC for 15 min, to remove non-specific binding. Sections were subsequently equilibrated for 10 min in PBS containing 0.1 % TritonX-100 (PBST), blocked with 10% goat serum in PBST buffer for 1 h, and then incubated with 1:1000 alkaline phosphatase (AP) conjugated anti-dig Fab fragment (Roche, Germany) in blocking solution overnight (ON) at 4°C. Finally, slides were washed in PBST, equilibrated in B3-Buffer (0.1 Tris-HCl, 0.1 M NaCl, 50 mM MgCl_2_, 0.1% Tween-20), followed by treatment with NBT/BCIP (Roche, Germany) (20 μl/ml B3) to visualize the hybridized probes.

### Immunohistochemistry (IHC)

After the fur and palate were removed, heads from adult male mice were fixed in 4% PFA at 4°C ON, then immersed in 30% sucrose at 4°C ON. 12 μm cryosections were prepared, blocked with 5% cold-water fish skin gelatine for 1 h at RT, and primary antibodies (1:250, Santa Cruz, CA, G_α_ olf sc-383; CNG sc-13700, ACIII sc-588, acetylated tubulin sc-23950) were applied in 1% cold-water fish skin gelatin in PBS containing 0.1% Triton X-100, at 4°C ON. After 30 min washing in PBS, secondary goat anti-rabbit antibodies Alexa Fluor 568 (Invitrogen, Germany) were applied for 30 min at RT in PBS. After 30 min washing in PBS, sections were embedded in ProlongGold Antifade (Invitrogen, Germany).

The Laird laboratory generously provided an antibody for Panx1 IHC (Penuela et al., 2007). For Panx1 detection the following modifications were introduced. For antigen retrieval, fixed cryostat sections were incubated for 5 min with 1% SDS, followed by three washes for 5 min with PBS. After blocking for 1 h at RT with 5% normal goat serum (NGS), 1% bovine serum albumin (BSA), and 0.1% Triton X100 in PBS, the primary antibody was diluted (1:100) in 1% BSA, 0.1% Triton X100 in PBS. After ON incubation, specimen were washed three times with PBS for 10 min each. The secondary goat anti-rabbit Alexa Fluor 488 antibody was diluted (1:1000) in PBS and applied for 30 min at RT, followed by three washes for 10 min with PBS.

Confocal microscopy was performed using a ZEISS LSM700 microscope. ZEISS ZEN software was used to control all parameters during imaging. Identical settings were used to allow a direct comparison of IHCs of Panx1^+/+^ and Panx1^−/−^ mice. LSM images were exported into tiff format and assembled using Photoshop CS.

### qPCR

RNA was isolated from adult male mice using the RNAeasy Fibrous Tissue Mini Kit (Invitrogen, Canada) and cDNA was synthesized from 1 μg total RNA with the ReadyScript cDNA Synthesis Kit (Sigma-Aldrich, Canada), according to the manufacturer's instructions. qPCR was performed using the SsoFast EvaGreen Supremix (Bio-Rad, Canada) and the following oligonucleotide pairs: Panx1fw: CAGGCTGCCTTTGTGGATTC Panx1rev: CGGGCAGGTACAGGAGTATG Panx2fw: GGTACCAAGAAGGCCAAGACT Panx2rev: GGGGTACGGGATTTCCTTCTC Panx3fw: CTTACAACCGTTCCATCCGC Panx3rev: CAGGTACCGCTCTAGCAAGG 18Sfw: TGACTCTTTCGAGGCCCTGTA 18Srev: TGGAATTACCGCGGCTGCTG. fw, forward; rev, reverse. Experiments were performed in triplicates, using three biological replicates. Relative gene expression was calculated using the REST software (2009) (Pfaffl et al., 2002). 18S served as the reference gene.

### EOG recordings

Skulls from adult male mice were cut parasagittal to the septum to expose the nasal cavity. The turbinates were removed and EOGs were recorded from the OE on the septum. A constant, deodorized, humidified air stream was delivered to the OE and adjusted to 2.4 l/min. Henkel 100 (H100, Henkel, Germany), a mixture of 100 different odors (Wetzel et al., 1999), was used as a stimulus. Dilutions were made in distilled water and soaked into a piece of felt, which was placed into a custom-made injection device. Recording electrodes were made from pulled glass capillaries containing chloride silver wires and filled with Ringer's solution (140 mM NaCl, 5 mM KCl, 1 mM CaCl_2_, 1 mM MgCl_2_, 10 mM HEPES, pH 7.4). During recording, heads were placed on 2% agarose dissolved in Ringer's solution containing the reference electrode. Student's *t*-test was used for statistical analysis.

### *Ex vivo* ATP assay

The intact OE was exposed after skulls from adult male mice were cut parasagittal to the septum to expose the nasal cavity. Once the OE was entirely exposed, the tissue was positioned in upside down orientation. Since the OE rapidly degenerates once axons get transsected, this *ex vivo* preparation was used instead of more invasive dissection procedures to extract extracellular ATP from the cilia surface. To avoid mechanical stimulation small droplets (25 μl) of Ringers solution, H100 [Henkel 100, 1:10000 in Ringers solution, Henkel, Germany (Wetzel et al., 1999)], and potassium gluconate (25 mM, Sigma-Aldrich, diluted in Ringers solution) were subsequently and gently pipetted onto the cilia surface at the center of the OE. The tissue was incubated for the indicated timespans. Between steps droplets were fully removed. Solutions contained 100 μM ARL 67156 trisodium salt hydrate (Sigma-Aldrich) to inhibit ATPases. Samples were heated at 95°C for 1 min after extraction, flash frozen, and stored at −80°C until used. ATP assays were performed in 96 well format; using the Molecular Probes® ATP Determination Kit (Life Technologies, USA) and the Synergy H4 hybrid multiwell plate reader (Biotek, USA). ATP concentrations were determined from ATP standard curves included in each assay. To test for statistical significance the student's *T*-test was used.

### Behavioral tests

#### Cookie-finding test

To familiarize the mice with the experimental setup, adult male mice were trained for 1 day to find a cookie (1 g) (Leibniz Butterkeks; Bahlsen, Germany) buried beneath 6 cm of woodchip bedding in their home cage. On the following days, smaller cookies of defined weight were hidden (see Results). The time to locate the cookie was determined from video recordings. We defined finding the cookie as the time point when mice held it in both fore paws. If a mouse did not find the cookie within 15 min, the test was aborted and most of the bedding above the cookie was removed, enabling the mouse to find the cookie very easily (usually within 1 min). Cookie-finding tests were analyzed using the *U*-test due to data truncation.

#### Mobility analysis

Animal mobility was analyzed with the EthoVision XT7 Software from Noldus (Wageningen, Netherland). Mobility was defined as distance per time. Tracking was stopped when the animal found the cookie. To test for statistical significance, Student's *T*-test was used.

## Results

### Pannexin expression and localization in the olfactory epithelium

**Figure 1:**
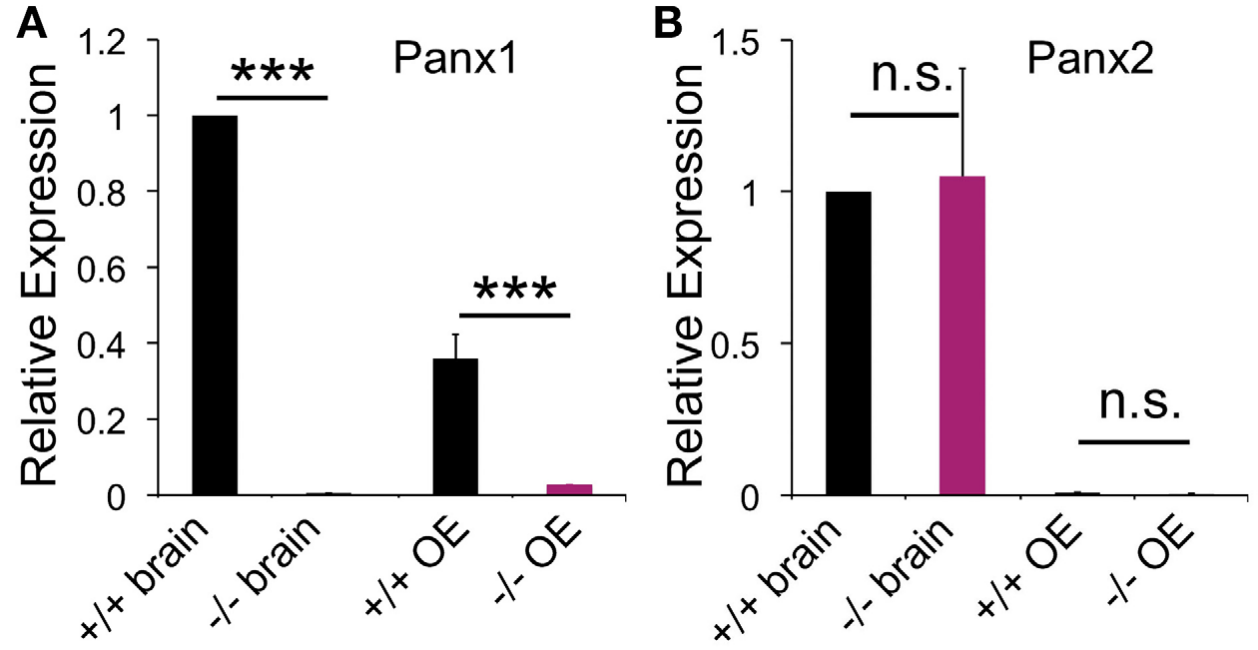
**Pannexin expression in the brain and OE.** qPCR data for pannexin expression in the OE (*N* = 3) and brain (*N* = 3) of adult mice. **(A)** Panx1^−/−^ mice lacked Panx1 expression. **(B)** No difference in Panx2 expression was detected in the OE. Expression of Panx3 was not detected. Primers specific for 18S were used as the reference. Experiments were performed in triplicates. ^***^p<0.001. n.s., not significant. Error bars: s.e.m.

Pannexin expression was determined in the mouse OE and whole brain lysate using qPCR and primer pairs specific for Panx1 (GI:86262133). Panx1 mRNA expression was found in the brain and OE adult mice (Figure 1), with mean cycle threshold (C_t_) values of 29.8 ± 0.5 (brain) and 31.3 ± 0.9 (OE). Panx2 was expressed in the OE at significantly lower expression levels compared to the brain (*p* < 0.001). Mean cycle threshold (C_t_) values were 27.2 ± 0.7 (brain) and 33.9 ± 0.7 (OE). No Panx3 expression was detected (*C_t_* < 35). Neither in the brain, nor in the OE a compensatory upregulation of Panx2 or Panx3 mRNA was observed in Panx^−/−^ mice. Consistent with the genetic phenotype, no Panx1 mRNA expression (*C_t_* < 35) was found in Panx1^−/−^ animals lacking exons 3 and 4 (Dvoriantchikova et al., 2012).

**Figure 2:**
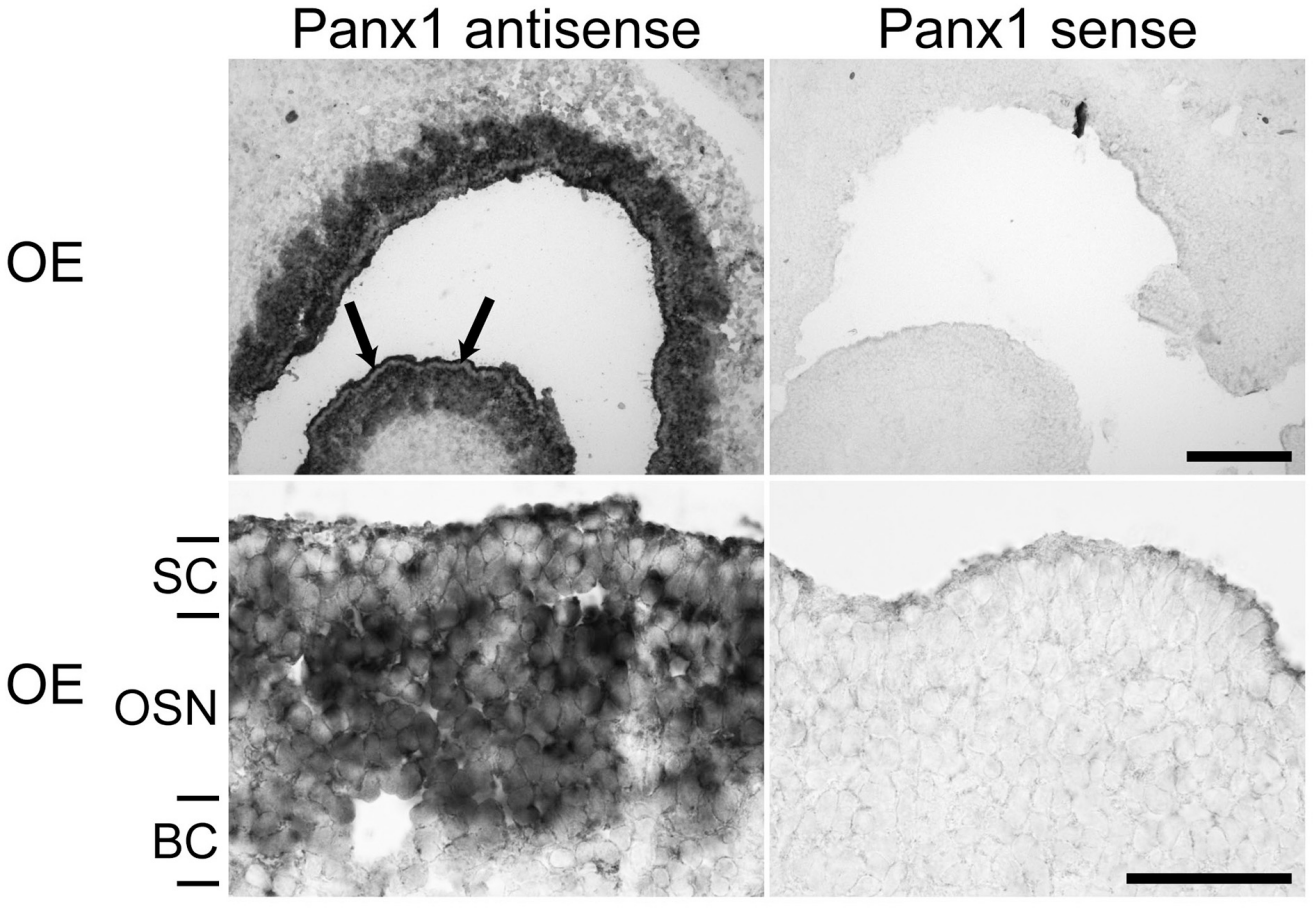
***In-situ* hybridizations with riboprobes specific for Panx1.** *In-situ* hybridizations from juvenile mice (P7) with Panx1 riboprobes. Panx1 antisense probes delivered strong labeling in the OE. Strong staining in the OE was present in the OSN layer. Weaker staining of SCs was more obvious in the overview (upper left, indicated by arrows). The BC layer showed weaker staining than the OSN layer. Scale bars = 200 μm, and 100 μm (close-up, middle) (OE, olfactory epithelium; SC, sustentacular cells; OSN, olfactory sensory neuron; BC, basal cell layer).

Localization of Panx1 expression sites in the OE were determined using *in-situ* hybridization technology and cRNA probes specific for mouse Panx1 (Ray et al., 2006). Figure 2 shows a representative distribution of Panx1 mRNAs in the OE. The sense cRNA probes generated only a very weak background staining, demonstrating the antisense probes' specificity. In the OE, a strong staining of the OSN layer and a less intense staining of the sustentacular (SC, arrows) and basal cell (BC) layers were observed.

**Figure 3:**
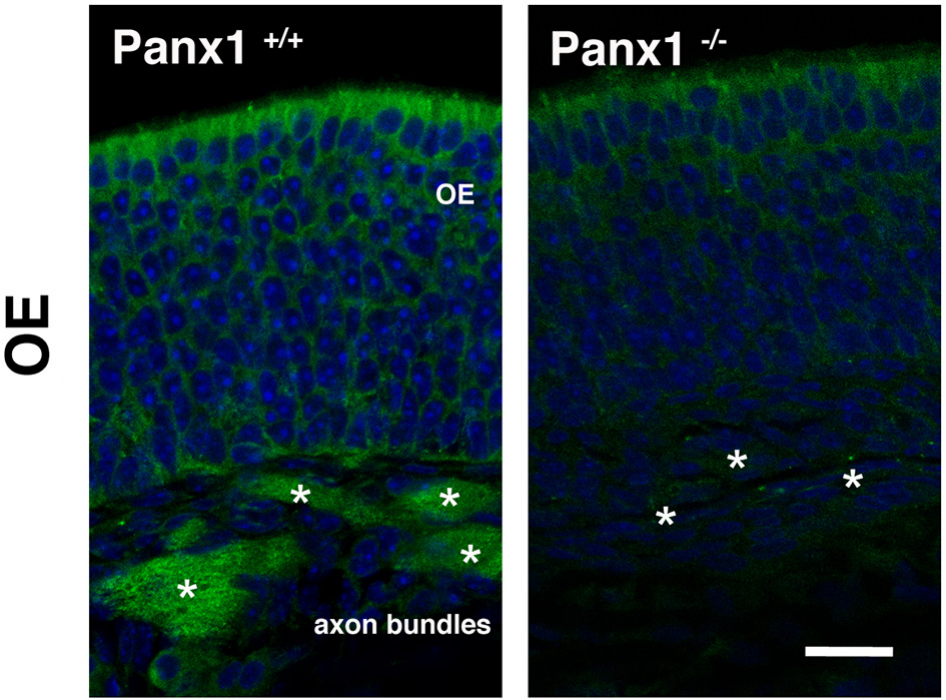
**Immunohistochemical detection of Panx1 in the OE.** IHC of histological section deriving from OE of adult Panx1^+/+^ and Panx^−/−^ mice. In the OE, a prominent Panx1 staining (in green) was identified in OE axon bundles (asterisks), which was not detected in Panx1^−/−^ mice. Nuclei were stained with DAPI (in blue). Images were recorded using identical conditions. Scale bars = 200 μm

To validate mRNA localization data, immunohistochemistry (IHC) was performed using an antibody specific for mouse Panx1 (Penuela et al., 2007). In Panx1^+/+^ animals (Figure 3), the IHC of the OE revealed a significant labeling of the OSN axon bundles, projecting to the olfactory bulb. Virtually no staining was found in OSN axon bundles of Panx1^−/−^ mice. However, a diffuse signal was observed in the basal-, neuronal-, and SC layers. No pronounced staining was found in OSN cilia, which inherit olfactory receptor proteins and signaling proteins involved in OSN signaling/depolarization after odorant activation.

### Odorant detection is not altered in Panx1^−/−^ mice

**Figure 4:**
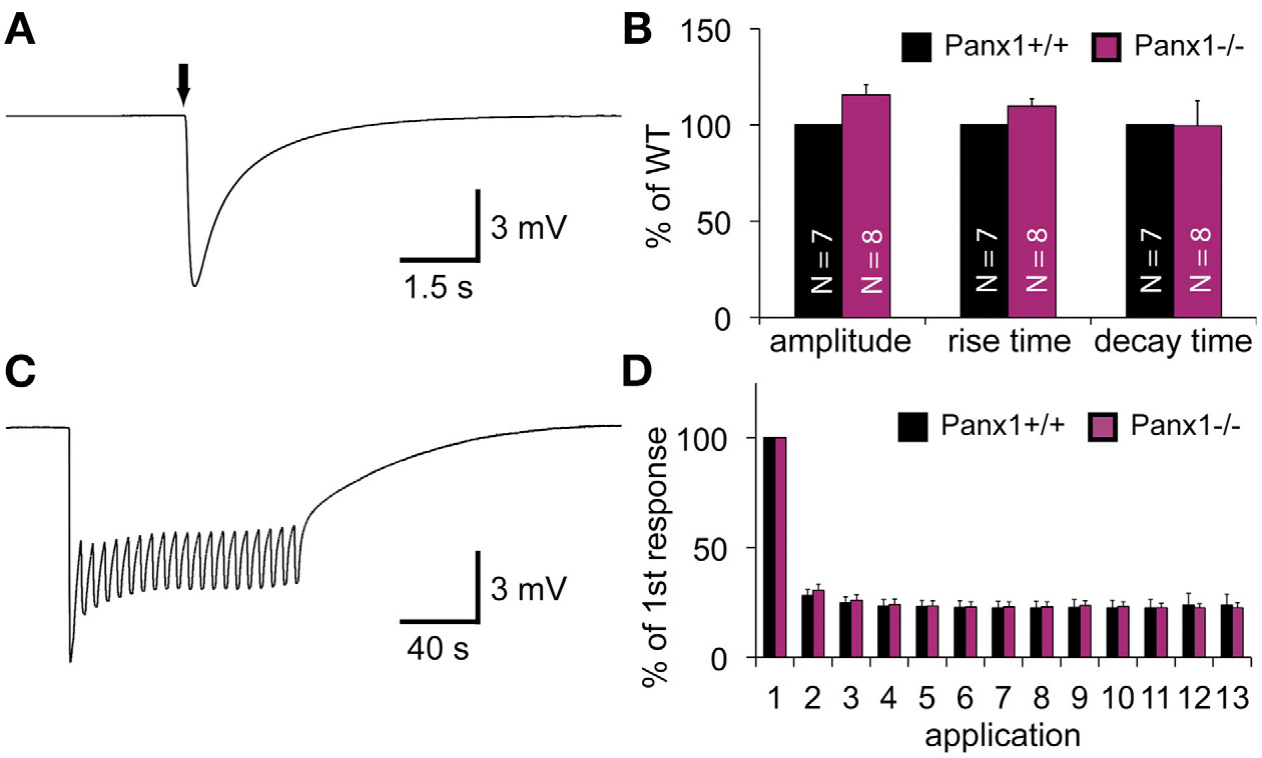
**EOG recordings of Panx1^+/+^ and Panx1^−/−^ olfactory epithelium. (A)** Sample EOG response after application of a single odorant pulse (100 ms; 1:1000 H100). **(B)** Quantification of single odorant pulses. Amplitude and response kinetics [rise (10–90%) and decay (90–10%) time] are not significantly different in Panx1^−/−^ mice (*p* > 0.05). Data were normalized to Panx1^+/+^ values. N_Panx1^+/+^_ = 7; N_Panx1^−/−^_ = 8; minimum 10 measurements per mouse. **(C)** Sample recording of multiple odor applications (1 s; 4 s interstimulus interval; 1:1000 H100). **(D)** Quantification of 13 adaptation measurements (depicted in **C**). Responses (Δ of individual responses) were normalized to the first response. No significant differences were found (N_Panx1^+/+^_ = 6; N_Panx1^−/−^_ = 6; 1 measurement per mouse). Error bars indicate s.e.m. (EOG, electroolfactogram; H100, Henkel 100).

When an olfactory receptor (OR) is activated, the signal transduction cascade elicited through the OR typically evokes a massive influx of calcium mediated by cyclic nucleotide gated (CNG) channels. Since Panx1 channels can be activated by raised intracellular calcium levels (Locovei et al., 2006), we investigated whether the lack of Panx1 alters the response of the OE upon odor application by performing electroolfactogram (EOG) measurements in adult male mice. A mixture of 100 different odorants [Henkel 100 (H100) (Wetzel et al., 1999)] was applied via a constant, humidified airstream to activate a broad range of ORs. Figure 4A shows a typical response amplitude after odorant application for 100 ms. Results were quantified and summarized in Figure 4B. No significant difference in the response amplitudes for Panx1^+/+^ (*N* = 7) and Panx1^−/−^ (*N* = 8) mice were detected (minimum 10 measurements on different locations per mouse, Panx1^+/+^ = 4.2 ± 0.2 mV, Panx1^−/−^ = 4.7 ± 0.3 mV, *p* = 0.17). Further, the response kinetics, namely the rise time (Panx1^+/+^ = 86 ± 3 ms, Panx1^−/−^ = 94 ± 4 ms, *p* = 0.1) and decay time (Panx1^+/+^ = 989 ± 191 ms, Panx1^−/−^ = 984 ± 128 ms, *p* = 0.98), did not differ between both animal groups.

To test the ability of the OE to adapt to odorants, we measured odor responses after a repetitive stimulation paradigm (Brunert et al., 2009) (N_Panx1^+/+^_ = 6, N_Panx1^−/−^_ = 6, 1 s stimulus duration, 4 s interstimulus interval, 1 measurement per mouse), as depicted in Figures 4C,D. Using this paradigm, we achieved a robust adaptation to less than 30 % of the control level, but adaptation kinetics observed in Panx1^−/−^ animals were statistically insignificant to those in wild type mice. Since Panx1^+/+^ and Panx1^−/−^ responded equally to both experimental conditions, we concluded that loss of Panx1 did not alter physiological odorant responses. Further confirming this finding, ablation of Panx1 did not cause a significant change in the localization and distribution of the four major olfactory signal transduction proteins detecting adenylyl cyclase 3 (ADCYIII), cyclic nucleotide gated channel alpha 2 (CNGA2), olfactory neuron specific-G protein (G_olf_), and acetylated tubulin (AcTub) (Supplementary Figure 1). Similarly, quantification of the mRNA expression of adenylyl cyclase 3 (ADCYIII), cyclic nucleotide gated channel alpha 2 (CNGA2), and olfactory neuron specific-G protein (G_olf_)using qPCR revealed no difference (Supplementary Figure 2).

### ATP release in the OE of Panx1^−/−^ mice

**Figure 5:**
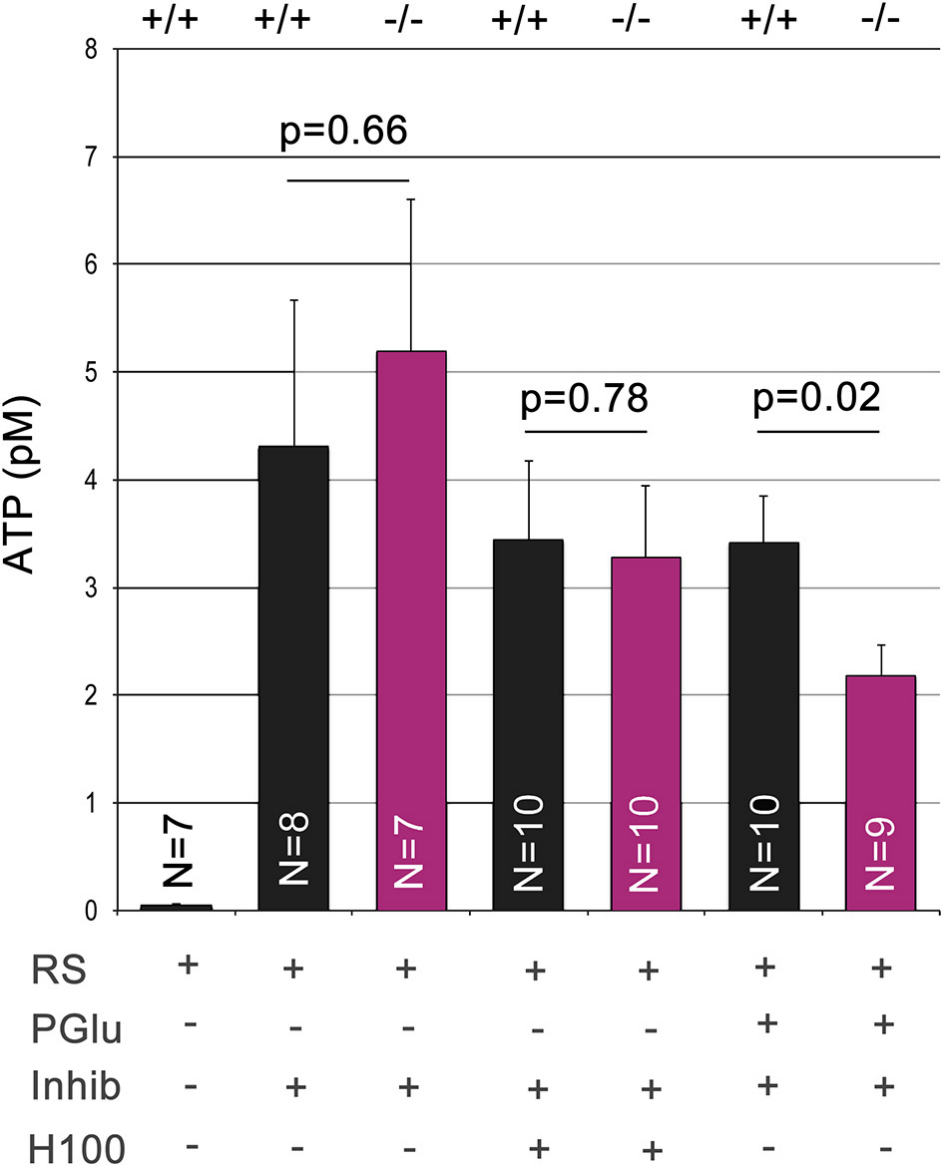
**Quantification of extracellular ATP.** Extracellular ATP was extracted from the ciliary surface of the OE after skulls from adult male mice were cut parasagittal to the septum to expose the nasal cavity. ATP concentrations were determined using this *ex vivo* preparation after serial application and complete extraction in small droplets containing Ringers solution (equilibration time: 3 min), Ringers solution with the ATPase inhibitor ARL 67156 (100 μM, equilibration time: 3 min), Henkel100 (equilibration time: 90 s) and finally potassium gluconate (25 mM, equilibration time: 90 s). Low levels of ATP did not differ in wild type and Panx1^−/−^ mice. Addition of the ATPase inhibitor increased ATP levels more than 100fold. Stimulation with Henkel100 caused significant ATP efflux in 90 s. Since no differences were detectable, we concluded that alternative ATP release pathways primarily promoted ATP efflux in response to the odorant. Subsequent stimulation with potassium gluconate for 90 s caused a reduction in ATP release in Panx1^−/−^ mice, demonstrating a significant role of Panx1 channels. Error bars indicate s.e.m.

Panx1 is considered to be a major ATP release channel in various tissues. We quantified extracellular ATP using *ex vivo* preparations of the OE (Figure 5). During this procedure the OE is placed upside down for extracellular ATP extraction. Due to the small size of the OE, ATP was extracted in a 25 μl droplet of Ringers solution gently placed at the center of the OE. After equilibration for 3 min, droplets recovered from the Panx1^+/+^ and Panx1^−/−^OEs had an ATP concentration in the pM range. Extracellular ATP concentrations in the 25 μl droplet increased more than 100fold when this extraction step was repeated with Ringers solution containing the ATPase inhibitor (ARL 67156). No significant difference was detected (*p* = 0.66), suggesting that ablation of Panx1 did not compromise efflux of extracellular ATP. After Ringers solution with ATPase inhibitor was completely removed, the odorant mixture Henkel 100 was applied for 90 s in a small droplet (25 μl) placed again at the center of the OE. No significant differences in extracellular ATP release in both mouse strains were detectable. The concentration of extractable ATP was slightly reduced to the previous step reflecting the reduced extraction time. It was concluded that alternative ATP release pathways primarily promoted the observed ATP efflux in response to the odorant. Subsequently 25 mM potassium gluconate in Ringers solution (25 μl) was applied for 90 s. Significant differences in extracellular ATP levels were detected (Panx1^+/+^, 3.4 ± 0.4 pM; Panx1^−/−^ 2.2 ± 0.3 pM; *p* = 0.022), suggesting that Panx1 channels contributed significantly to ATP release in this experimental condition.

### Odorant perception in Panx1^−/−^ mice

**Figure 6:**
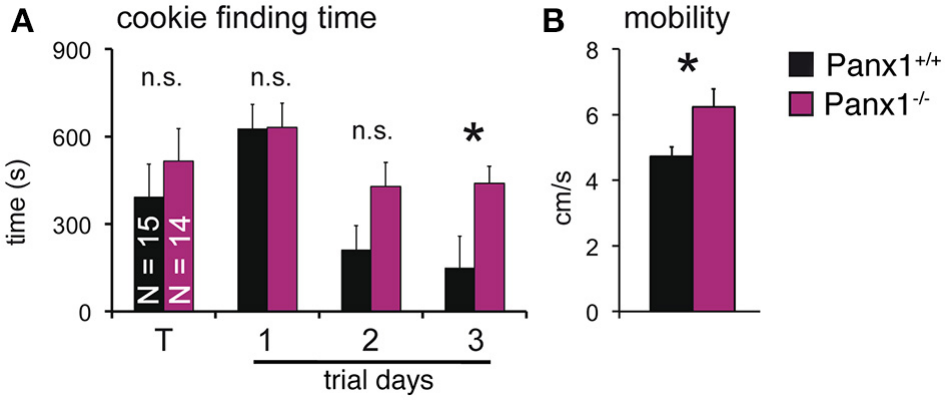
**Cookie-finding test. (A)** Latency to find a hidden cookie on four trials. Cookie sizes used: Training (T) trial: 1 g; trial 1: 50 mg; trial 2: 150 mg; trial 3: 50 mg. Panx1^−/−^ mice took significantly longer on trial 3 to retrieve the cookie (*p* = 0.02, N^−/−^_Panx1_ = 14, N^+/+^_Panx1_ = 15). **(B)** Velocity analysis showed a significantly higher mobility of Panx1^−/−^ mice. ^*^p<0.05. Error bars indicate s.e.m.

The results shown suggested that loss of Panx1 does not impair detection of odorants in the OE. However, Panx1 is expressed in other cells of the olfactory system, including subsets of neurons in the olfactory bulb and the piriform cortex (Bruzzone et al., 2003; Ray et al., 2005). To test for odorant perception, a cookie-finding test was performed. In this behavioral test, an odorous cookie was hidden beneath the bedding in the home cage of each mouse and the latency until the mice found the treat was determined. Tests were conducted on four subsequent days (Figure 6A). On the training day (T), a large cookie (1 g) was hidden. The size (and odor) allowed the mice to familiarize with the test. Panx1^+/+^ (*N* = 15) and Panx1^−/−^ (*N* = 14) mice performed equally on test day 1, when test conditions were more difficult for the mice (50 mg cookie; Panx1^+/+^ = 627 ± 83 s, Panx1^−/−^ = 630 ± 82 s). We performed two more tests using a large [150 mg (day 2)] and a small [50 mg (day 3)] cookie. On day 2, Panx1^+/+^ animals showed a tendency to find the cookie faster than Panx1^−/−^ mice, however, this difference was not significant (Panx1^+/+^ = 212 ± 83 s, Panx1^−/−^ = 428 ± 82 s, *p* = 0.1). When the difficulty of the test was increased on day 3 (50 mg cookie), Panx1^−/−^ mice took significantly longer to find the cookie (Panx1^+/+^ = 149 ± 58 s, Panx1^−/−^ = 439 ± 107 s, *p* = 0.02). Further, they did not improve compared to the previous days (day 2) and training day (T) when conditions were easier (Figure 6A). This suggests that processing of olfactory information was not affected, as both animal cohorts did find the 50 mg cookie on test day one at the same time, but the ability of the animals to learn the test was significantly altered. We also tracked the velocity of the animals and found that Panx1^−/−^ mice showed a significantly higher mobility (Panx1^+/+^ = 4.7 ± 0.3 cm/s, Panx1^−/−^ = 6.2 ± 0.5 cm/s, *p* = 0.02) compared to Panx1^+/+^ mice (Figure 6B).

## Discussion

Our results show that Panx1 and Panx2 are expressed in the OE, with the expected lack of Panx1 expression in Panx1^−/−^ mice. No compensatory regulation of Panx2 was observed. *In-situ* hybridizations of OE slices revealed that Panx1 mRNA is expressed in OSNs, and to a lower extent in SCs and basal cells, which is in accordance to a recent study (Zhang et al., 2012). We further showed that the Panx1 protein is localized in axon bundles of OSNs. The role of Panx1 channels in axon bundles is unclear. However, this localization and the apparent lack of Panx1 channels in the ciliary layer of the OE could explain why Panx1 is not directly involved in the olfactory signal transduction and activation of OSNs, in which case a ciliary localization would be most likely. Stimulation with short, single odorant pulses, or testing of adaptation, did evoke similar responses in Panx1^−/−^ and Panx1^+/+^ mice in EOG recordings. The kinetics of the EOG responses, namely the rise and decay times, were similar in both mouse groups, indicating no major difference in signal amplification and termination mechanisms, further substantiated by IHC staining and qPCR data for major signal transduction proteins, which showed no significant changes in localization and steady state mRNA expression (Supplementary Figures 1, 2), Behavioral testing was used to investigate the animals' capability to detect odorant cues. Since processing of olfactory information appeared unaffected, whereas the ability of the animals to learn the test was significantly altered, we conclude that learning and memory capabilities are impaired in Panx1^−/−^ animals (Prochnow et al., 2012).

Extracellular ATP is known to modulate olfactory responsiveness and influence proliferation and neuronal differentiation (Hassenklöver et al., 2009; Jia et al., 2009, 2011; Gao et al., 2010). It was tempting to hypothesize that the ablation of Panx1, a major release channel for ATP (Locovei et al., 2006; Qiu and Dahl, 2009; Dahl and Keane, 2012), alters OE function, either by influencing neuronal turnover in the OE, or being directly involved in the olfactory signal transduction/adaptation. Therefore, we quantified extracellular ATP concentrations *ex vivo* in the OEs of unchallenged Panx1^+/+^ and Panx1^−/−^ animals, and after exposure to H100 and potassium gluconate. The latter has previously been shown to open Panx1 channels *in vitro* (Silverman et al., 2009). Application of potassium gluconate revealed significant changes between wild type and Panx1^−/−^ animals, with ablation of Panx1 channels causing a significant reduction of potassium gluconate stimulated ATP release. The observed reduction is consistent with two previous studies. Similar to our results, extracellular ATP deriving from isolated erythrocytes (Qiu et al., 2011), or lumbar and sacral spinal cord slices (Lutz et al., 2013) were virtually identical in Panx1^+/+^ and Panx1^−/−^ mice. In contrast to human erythrocytes, where Panx1 channel inhibitors almost completely abolish ATP release, a substantial release remained in mouse erythrocytes even after probenecid treatment and the reduction in ATP release upon potassium stimulation was less than 50% (Qiu et al., 2011). Taken together, these findings are remarkable consistent in two independently generated Panx1KO models, supporting the role of Panx1 in ATP release and pointing at the existence of alternative ATP release pathways.

A challenge in future investigations will be to dissect the alternative ATP release pathways. Candidates are manifold and include vesicular release, connexin hemichannels, and ABC transporters, all present in the OE (Hayoz et al., 2012). In this study, we provide first evidence that LRRC8 channels, recently described as proteins with structural similarities to connexins and pannexins (Abascal and Zardoya, 2012), are expressed in the OE (Supplementary Figure 3). However, it remains to be demonstrated whether these channels are capable of releasing ATP. It is worth noting that expression of CALHM1, another channel with structural similarities to pannexins and ATP release capability (Siebert et al., 2013; Taruno et al., 2013) was not detected in the OE (unpublished data). Finally, compensatory regulation of connexins or pannexin proteins (Lohman and Isakson, 2014; Penuela et al., 2014) needs to be investigated to clarify synergistic or competing mechanisms of ATP efflux without Panx1 channels.

Arguably, the results presented in this study strongly advocate for a detailed analysis of ATP release mechanisms in the absence of Panx1. As shown, the characterization of Panx1^−/−^ mice does not support a prominent role of Panx1 channels in olfaction, and it was concluded that the behavioral abnormalities observed in Panx1^−/−^ mice derive from alterations of integrating neuronal processes, as observed in the hippocampus. In summary, our findings highlight the role of Panx1 as a major ATP release site operating in the OE alongside alternative pathways.

## Author contributions

Stefan Kurtenbach and Georg Zoidl planned the project. Stefan Kurtenbach, Paige Whyte-Fagundes, Lian Gelis, Christiane Zoidl performed experiments. Stefan Kurtenbach, Georg Zoidl wrote the manuscript. Sarah Kurtenbach, Valery I. Shestopalov, and Hanns Hatt helped with data evaluation, interpretation, and manuscript preparation. Valery I. Shestopalov generated the mouse model.

### Conflict of interest statement

The authors declare that the research was conducted in the absence of any commercial or financial relationships that could be construed as a potential conflict of interest.
