# Supplementary material for: Investigation of olfactory function in a Panx1 knock out mouse model
Source: Front Cell Neurosci. 2014 Sep 12;8:266. doi: 10.3389/fncel.2014.00266 (PMC4162419; doi:10.3389/fncel.2014.00266)
Supplement: Supplementary file 1 [file Presentation1.PDF]

Supplementary Methods:

qPCR of olfactory pathway gene expression

Total RNAs were isolated from adult male mice using the RNAeasy Fibrous Tissue Mini Kit (Invitrogen, Canada). cDNAs were synthesized from 1µg total RNA with the ReadyScript cDNA Synthesis Kit (Sigma-Aldrich, Canada), according to the manufacturer's instructions. qPCR was performed using the SsoFast EvaGreen Supremix (Bio-Rad, Canada) and the following oligonucleotide pairs: 18Sfw: 5'-TGACTCTTTCGAGGCCCTGTA-3'; 18Srev: 5'-TGGAATTACCGCGGCTGCTG-3'. Cnga2 RefSeq ID: NM_007724. CNGA2fw: 5'-AGCTCTTCACACCGTTGGAT-3', CNGA2rev: 5'- CAACAGATGCTGACTTTGGC-3'. Amplicon size: 103bp. Adcy3, RefSeq ID: NM_138305. ADCY3fw 5'-ACATGATGCCCACGATGATA-3', ADCY3rev 5'-CAGCAGGATGAGCTGGAAG-3'. Amplicon size: 91bp. Gnal (G_{olf}), RefSeq ID: NM_010307. GNAIfw 5'-CTTCATCGTCCCACAGCTTC-3', GNAIrev 5'-GAGAACCAGTTCCGGTCAGA-3'. Amplicon size: 106bp. All amplicons span exon to intron boundaries. No primers were generated to test for acetylated tubulin. Experiments were performed in triplicates, using three (OE and brain) biological replicates. All experiments were performed using the Eppendorf Mastercycler Gradient (Eppendorf, Canada). Relative gene expression was calculated using the REST software (Pfaffl et al., 2002) 18S served as the reference gene.

RT-PCR detection of LRRC8 and CALHM1 mRNA expression

Total RNAs were isolated and processed to cDNA as described above. Detection of the mouse calcium homeostasis modulator 1 (Calhm1; GI:124486908), and the leucine rich repeat containing 8A (Lrrc8a; GI:227908848) steady state RNA expression was determined by endpoint

PCR using HotStarTaq *Plus* DNA Polymerase (Qiagen, Canada), the Eppendorf Mastercycler Gradient (Eppendorf, Canada). Cycling conditions were: Hold at 95°C for 15 minutes before cycling; cycle for 30 seconds at 94°C, 30 seconds at 55°C and 30 seconds at 72°C for a total of 35 cycles. PCR products were detected using agarose gel electrophoresis. Primers were: 18Sfw, 5'-TGACTCTTTCGAGGCCCTGTA-3', 18Srev 5'-TGGAATTACCGCGGCTGCTG-3', LRRC8Afw 5'-TCGAGAAAATCCCCACCCAG-3', LRRCA8rev 5'-GTGTAGAGCCCGTAGCTTCC-3', LRRC8Bfw, 5'-GCAGAGTTTCCGGTTGACCA-3', LRRC8Brev 5'-GGTAGGATGACTGGGCGTC-3', LRRC8Cfw 5'-GGCATCCTTTTCTGCGGATAC-3', LRRC8Crev 5'-CCACCGAGAGGTAATCCGTG-3', LRRC8Dfw 5'- CACTGCTGACTGCAACGTG-3', LRRC8Drev 5'-CCATGGCTTCAGGATTCGGT-3', LRRC8Efw 5'-GCGGAGTTCAAGCAGTTCAC-3', LRRC8Erev 5'-GGAGCCCCCGATATGTTCTC-3'. mCALHM1fw 5'-TCCAGAACTTGCTCGCCTAC-3', mCALHM1rev 5'-TCTTGAGAAAGGCGACCTGC-3'.

Immunohistochemistry (IHC) of OE marker proteins

After the fur and palate were removed, heads from adult male mice were fixed in 4 % PFA at 4°C ON, then immersed in 30 % sucrose at 4°C over night. 12 µm cryosections were prepared, blocked with 5 % gelatine for 1 h at RT, and primary antibodies (1:250, Santa Cruz, CA, G_{α}olf sc-383; CNG2 sc-13700, ACIII sc-588, acetylated tubulin sc-23950) were applied in 1 % cold-water fish skin gelatin in PBS containing 0.1 % Triton X-100, at 4°C ON. After 30 min washing in PBS, secondary goat anti-rabbit antibodies Alexa Fluor 568 (Invitrogen, Germany) were applied for 30 min at RT in PBS. After 30 min washing in PBS, sections were embedded in ProlongGold Antifade (Invitrogen, Germany). Confocal microscopy was performed using a

ZEISS LSM700 microscope. ZEISS ZEN software was used to control all parameters during imaging. Identical settings were used to allow a direct comparison of IHCs of Panx1 $^{+/+}$ and Panx1 $^{-/-}$ mice. LSM images were exported into tiff format and assembled using Photoshop CS.

Pfaffl, M.W., Horgan, G.W., and Dempfle, L. (2002). Relative expression software tool (REST) for group-wise comparison and statistical analysis of relative expression results in real-time PCR. *Nucleic Acids Res* 30**,** e36.

Supplementary Figure Legends

Supplementary Figure 1 – *Immunohistochemical stainings of Panx1$^{+/+}$ and Panx1$^{-/-}$ olfactory epithelium reveals no difference in odorant signal transduction proteins*

The ciliary region of the mouse (P21) olfactory epithelium is intensely stained with antibodies detecting adenylyl cyclase 3 (ADCYIII), cyclic nucleotide gated channel alpha 2 (CNGA2), olfactory neuron specific-G protein ($G_{olf)}$ and acetylated tubulin (AcTub). The proteins selected served as ciliary marker and markers of odorant transduction in the OE. No difference of fluorescence distribution and intensity is found comparing Panx1$^{+/+}$ and Panx1$^{-/-}$ mice when images were collected using identical settings. Scale bars = 40 µm. (ACIII = adenylyl cyclase type III; CNGA2 = cyclic nucleotide gated channel A2 subunit, G_{olf} = olfactory G-Protein, AcTub = acetylated tubulin).

Supplementary Figure 2 – ***Quantification of steady state mRNA expression of odorant transduction proteins and markers for cilia***

The quantification of adenylyl cyclase 3 (ADCYIII), cyclic nucleotide gated channel alpha 2 (CNGA2) and olfactory neuron specific-G protein (GnaI/G_{olf}) mRNA expression in the OE (N = 3) of wild type and Panx1$^{-/-}$ mice showed no significant differences in cycle threshold (C_t), in line with the immunohistochemical stainings. Primers specific for 18S were used as a control. Experiments were performed in triplicates. N.S., not significant. Error bars: S.E.M

Supplementary Figure 3 – ***Expression of LRRC8 channels in the OE***

The mRNA expression of Leucine-rich repeat-containing 8 (LRRC8) mRNA variants A-E and the calcium homeostasis modulator 1 (CALHM1) in the OE was tested using endpoint RT-PCR. The amplicon produced by 18s rRNA RT-PCR was 100 bp, while the amplicons produced by the LRRC8 A, B, C, D and E paraloges were ~200 bp, ~210 bp, ~210 bp, ~210 bp, and ~190 bp, respectively. No mRNA expression of CALHM1 was detected, which was consistent with the lack of expression in neuronal tissues reported in public human and mouse RNAseq databases (GEO-Profiles, National Center Biotechnology Information). The results shown suggest that LRRC8 mRNA is expressed in the OE and further testing is needed to determine whether this channel can complement Panx1 functions in wild type and knock out mice.
