# Supplementary figures and images for: Investigation of olfactory function in a Panx1 knock out mouse model
Source: Front Cell Neurosci. 2014 Sep 12;8:266. doi: 10.3389/fncel.2014.00266 (PMC4162419; doi:10.3389/fncel.2014.00266)

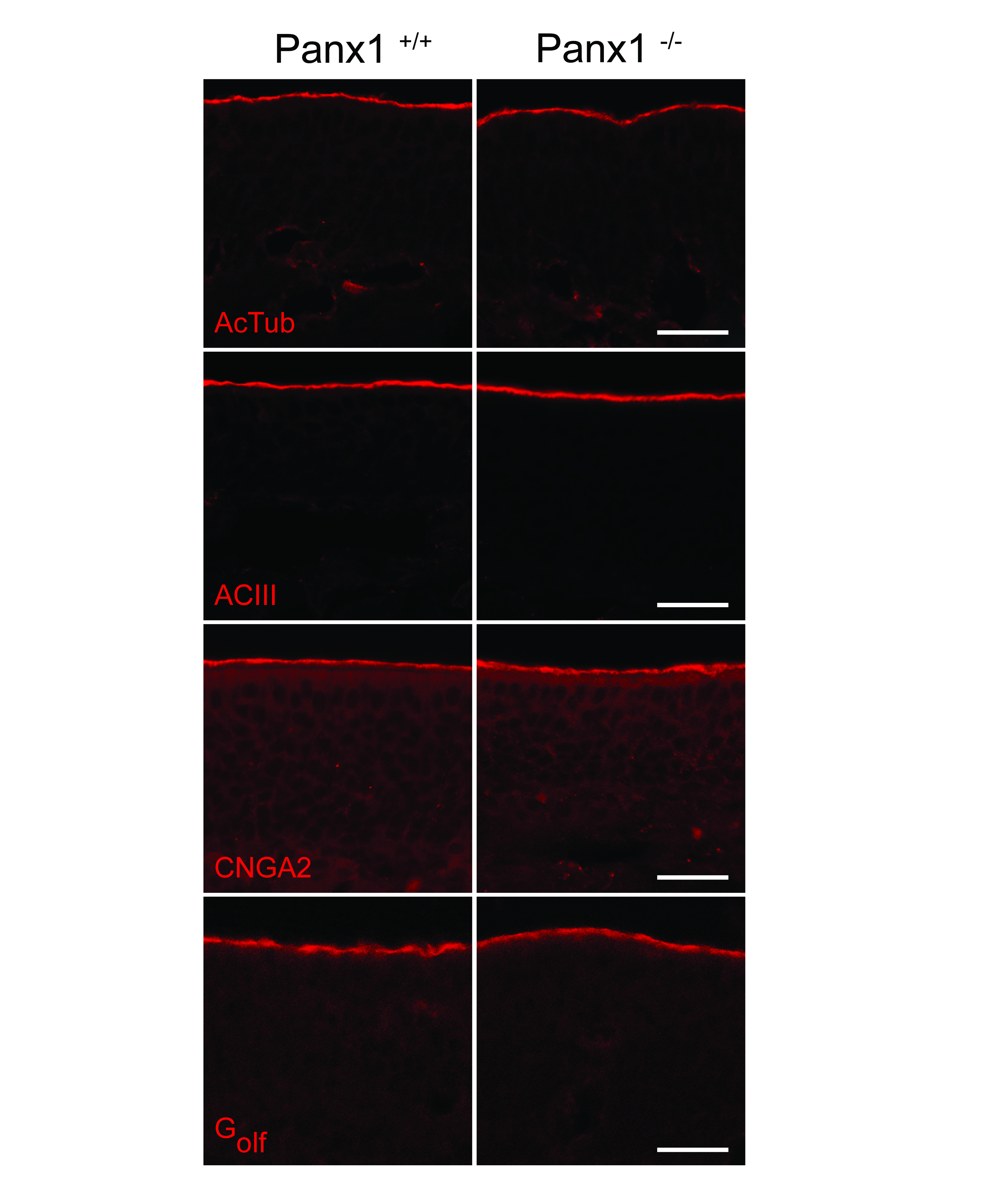

Supplement: Supplementary file 2 [file Image1.TIF]

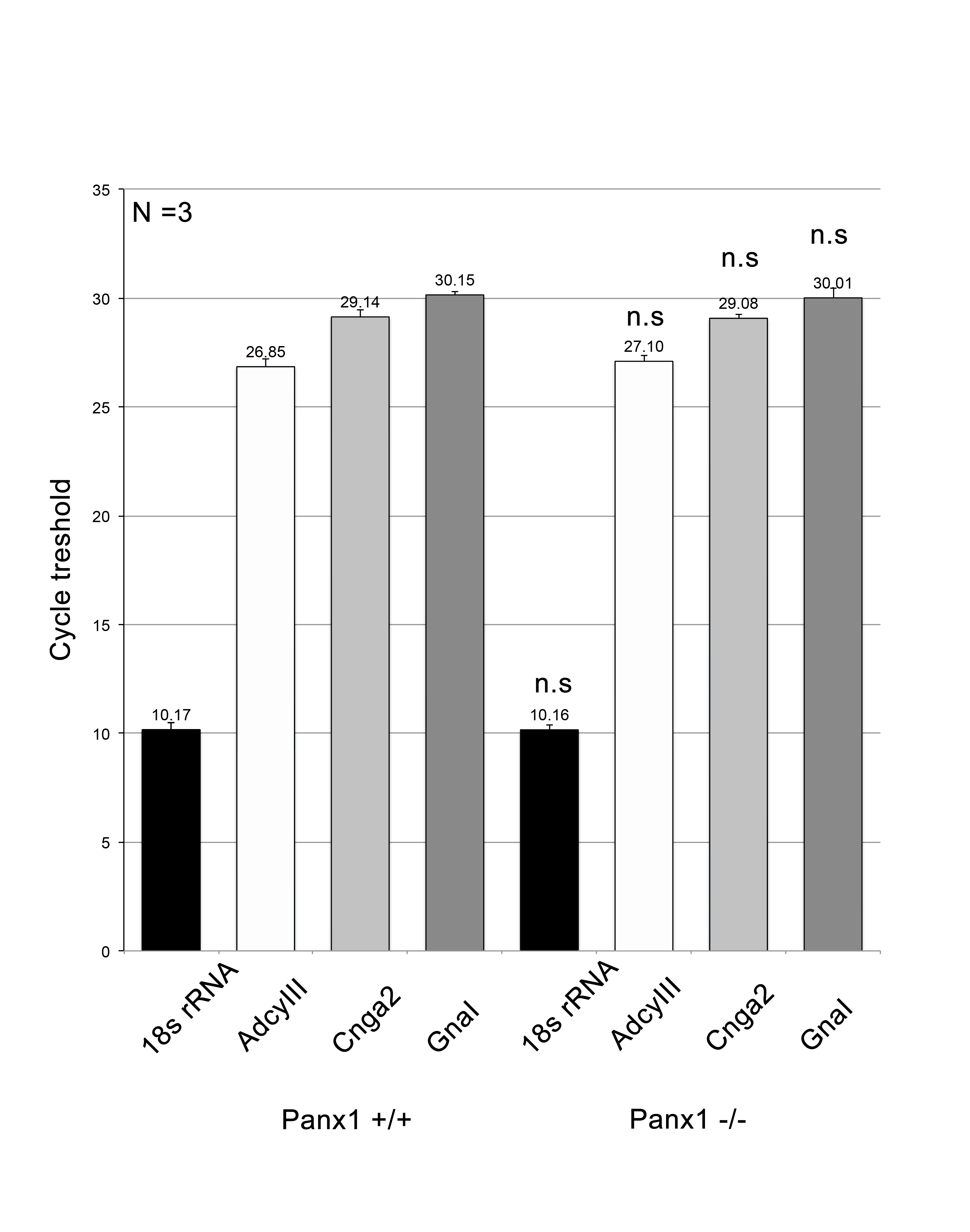

Supplement: Supplementary file 3 [file Image2.TIF]

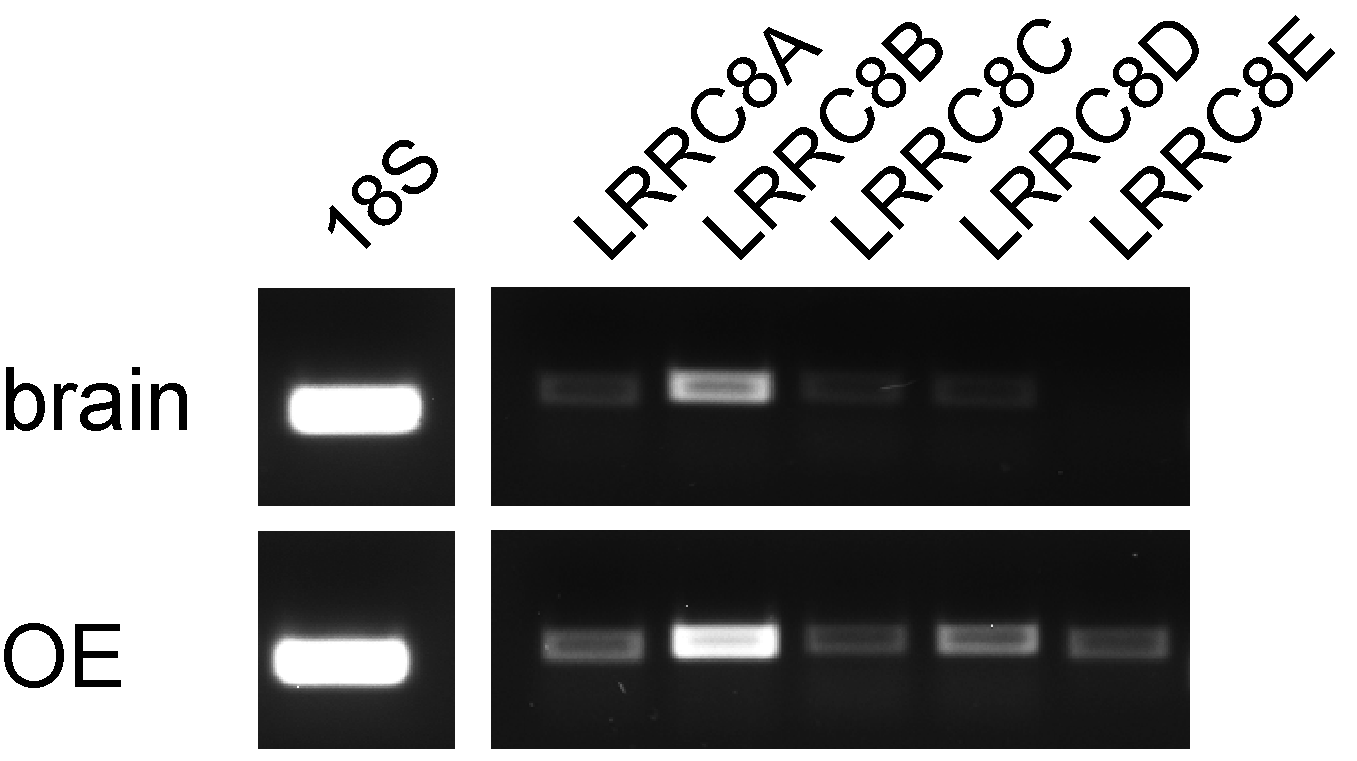

Supplement: Supplementary file 4 [file Image3.TIF]
